# Supplementary figures and images for: PICH impacts the spindle assembly checkpoint via its DNA translocase and SUMO-interaction activities
Source: Life Sci Alliance. 2025 Feb 7;8(4):e202403140. doi: 10.26508/lsa.202403140 (PMC11806350; doi:10.26508/lsa.202403140)

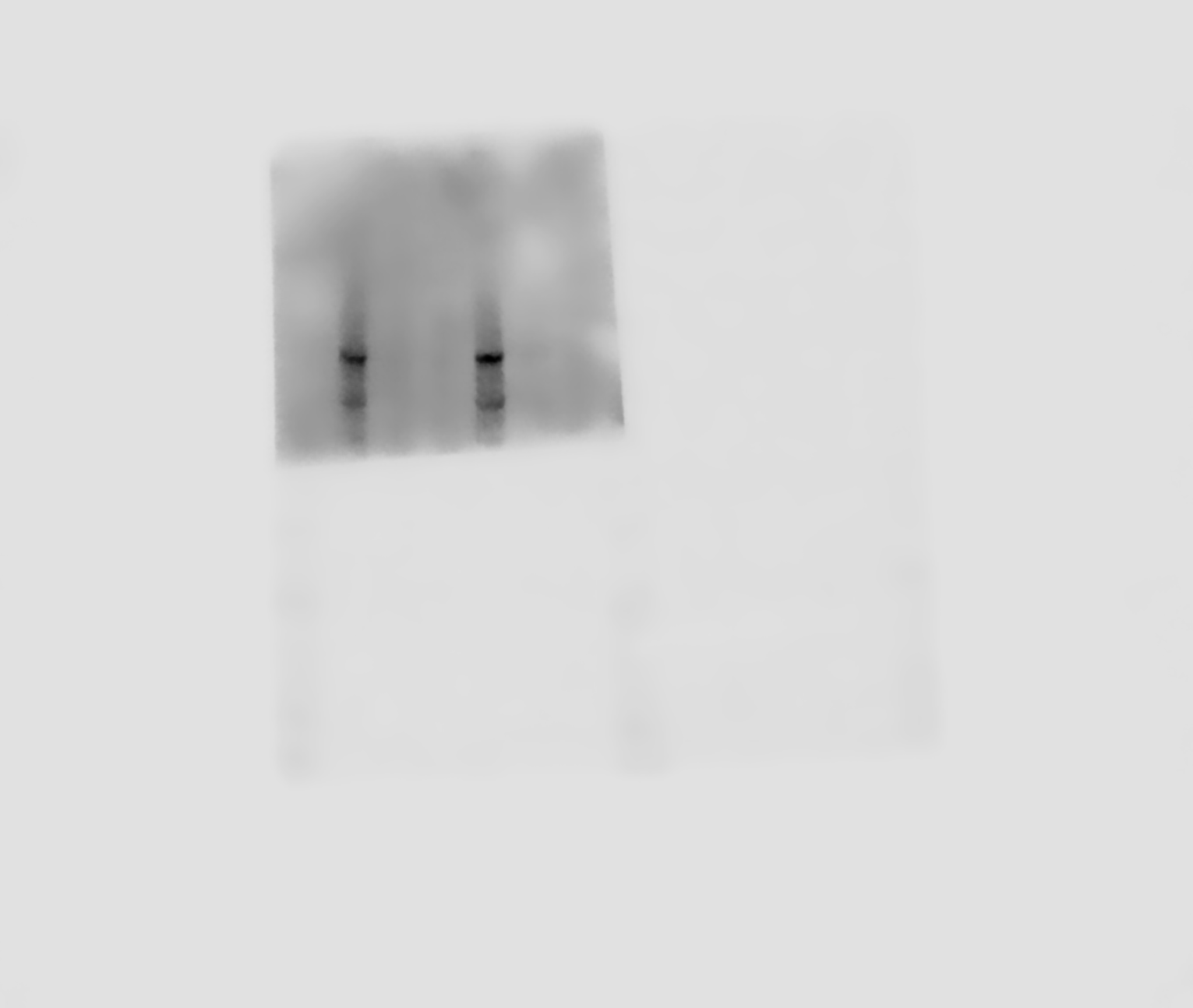

Supplement: Supplementary file 1 [file LSA-2024-03140_SdataF1_F2_F4.4.zip › western blot file-LSA/fig 2A PICH.tif]

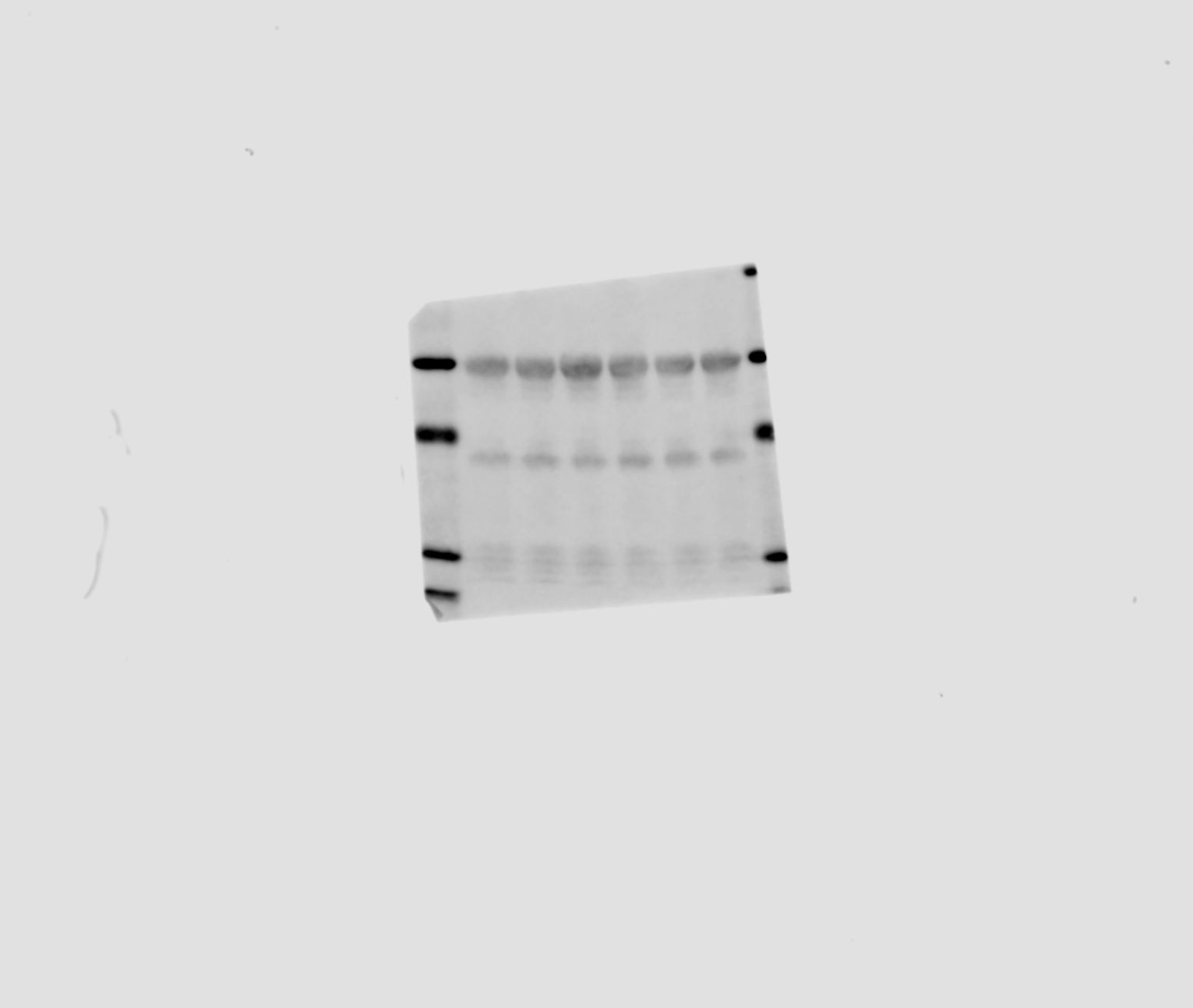

Supplement: Supplementary file 1 [file LSA-2024-03140_SdataF1_F2_F4.4.zip › western blot file-LSA/fig 2A tubulin.tif]

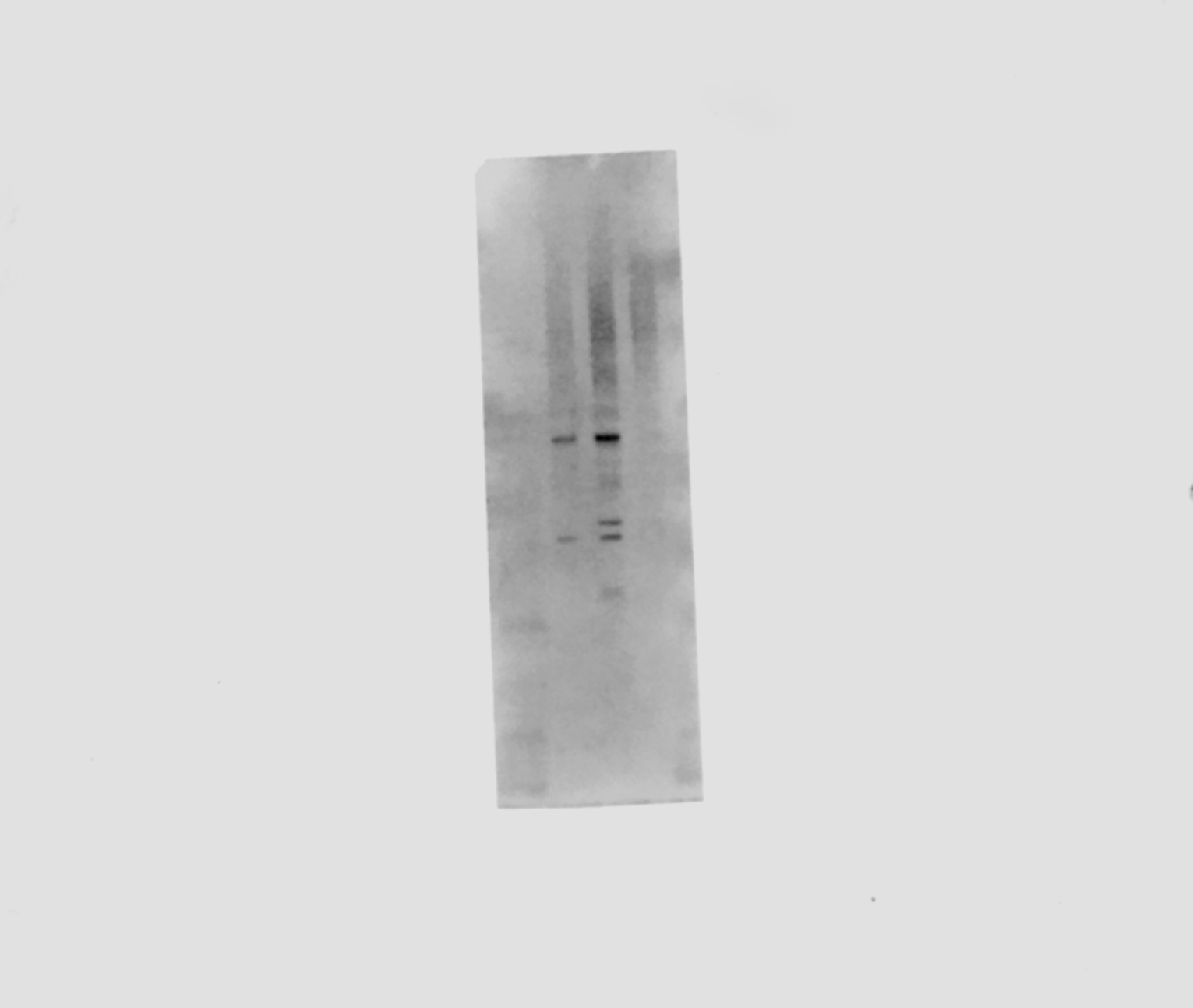

Supplement: Supplementary file 1 [file LSA-2024-03140_SdataF1_F2_F4.4.zip › western blot file-LSA/fig 4D _his6 purified .tif]

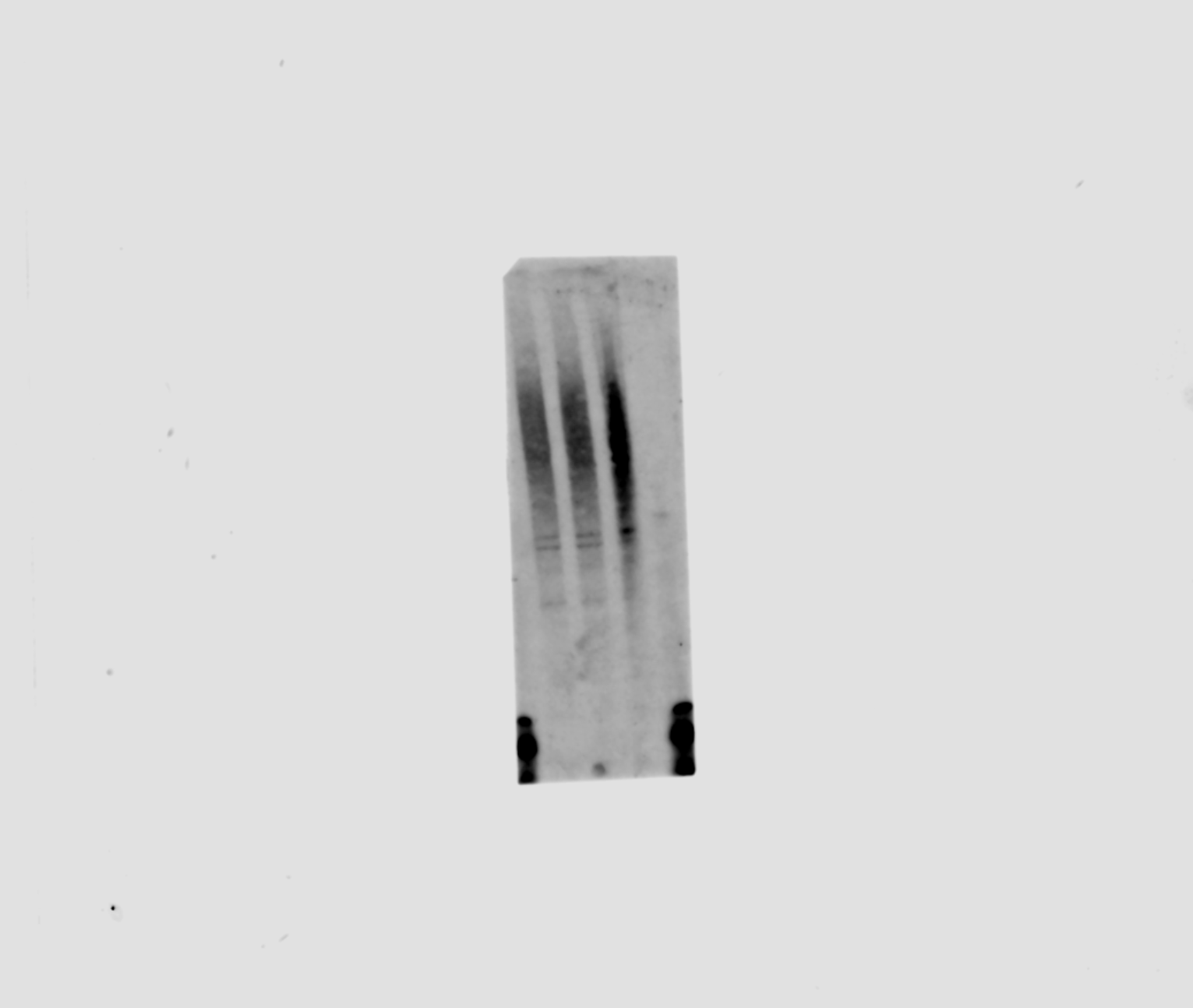

Supplement: Supplementary file 1 [file LSA-2024-03140_SdataF1_F2_F4.4.zip › western blot file-LSA/fig 4C _sumo.tif]

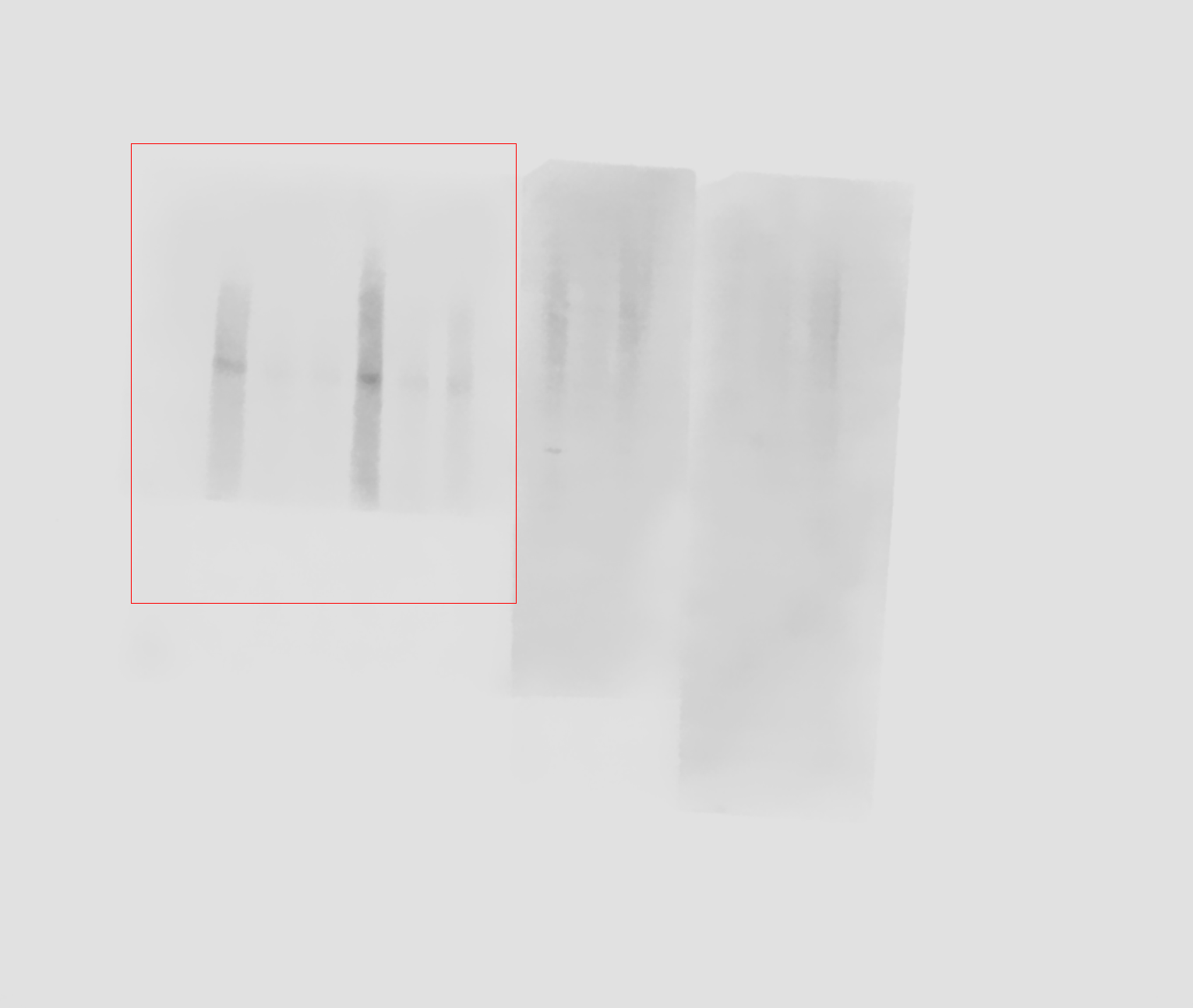

Supplement: Supplementary file 1 [file LSA-2024-03140_SdataF1_F2_F4.4.zip › western blot file-LSA/fig 2A _flag.tif]

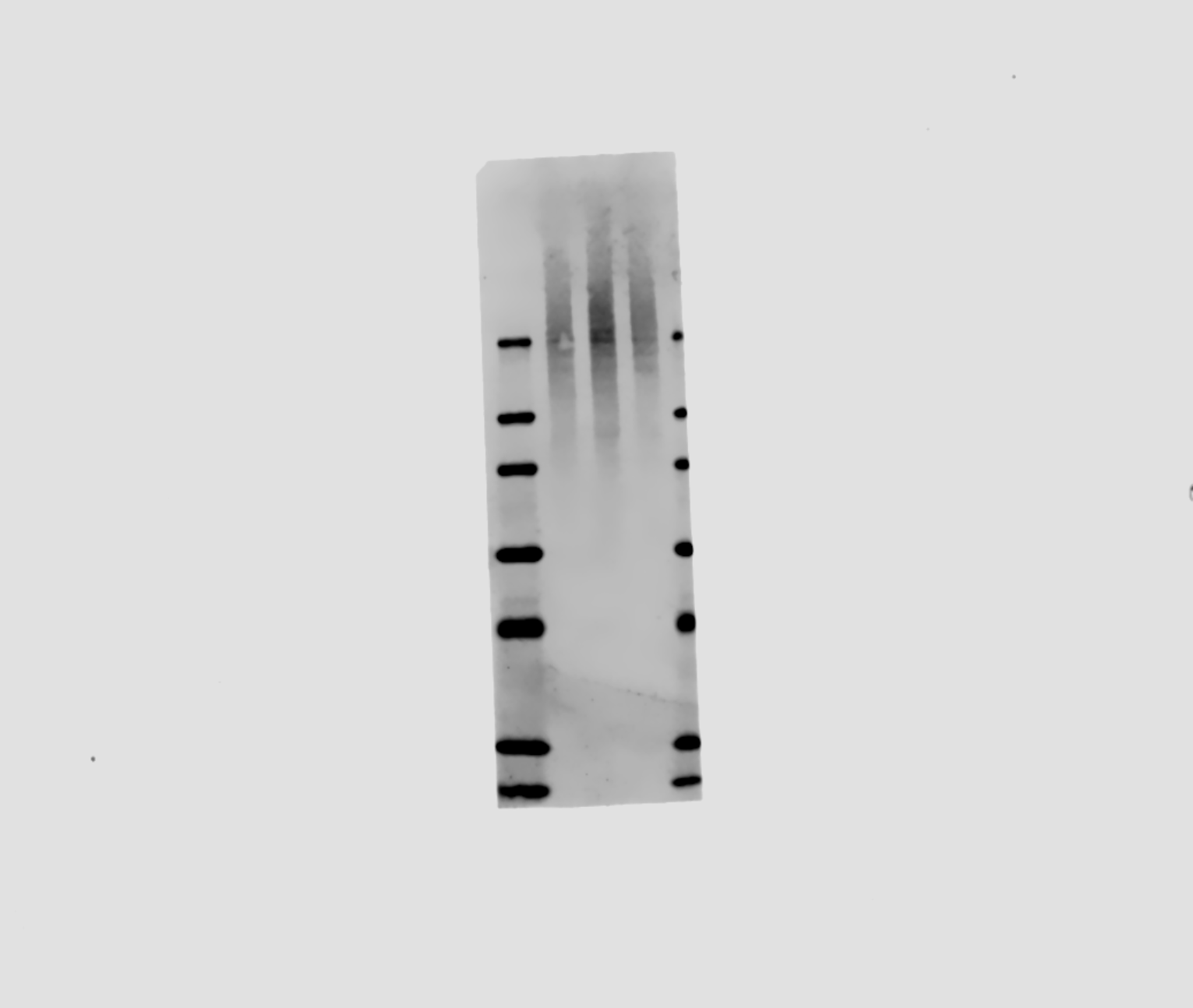

Supplement: Supplementary file 1 [file LSA-2024-03140_SdataF1_F2_F4.4.zip › western blot file-LSA/fig 4D _sumo purified .tif]

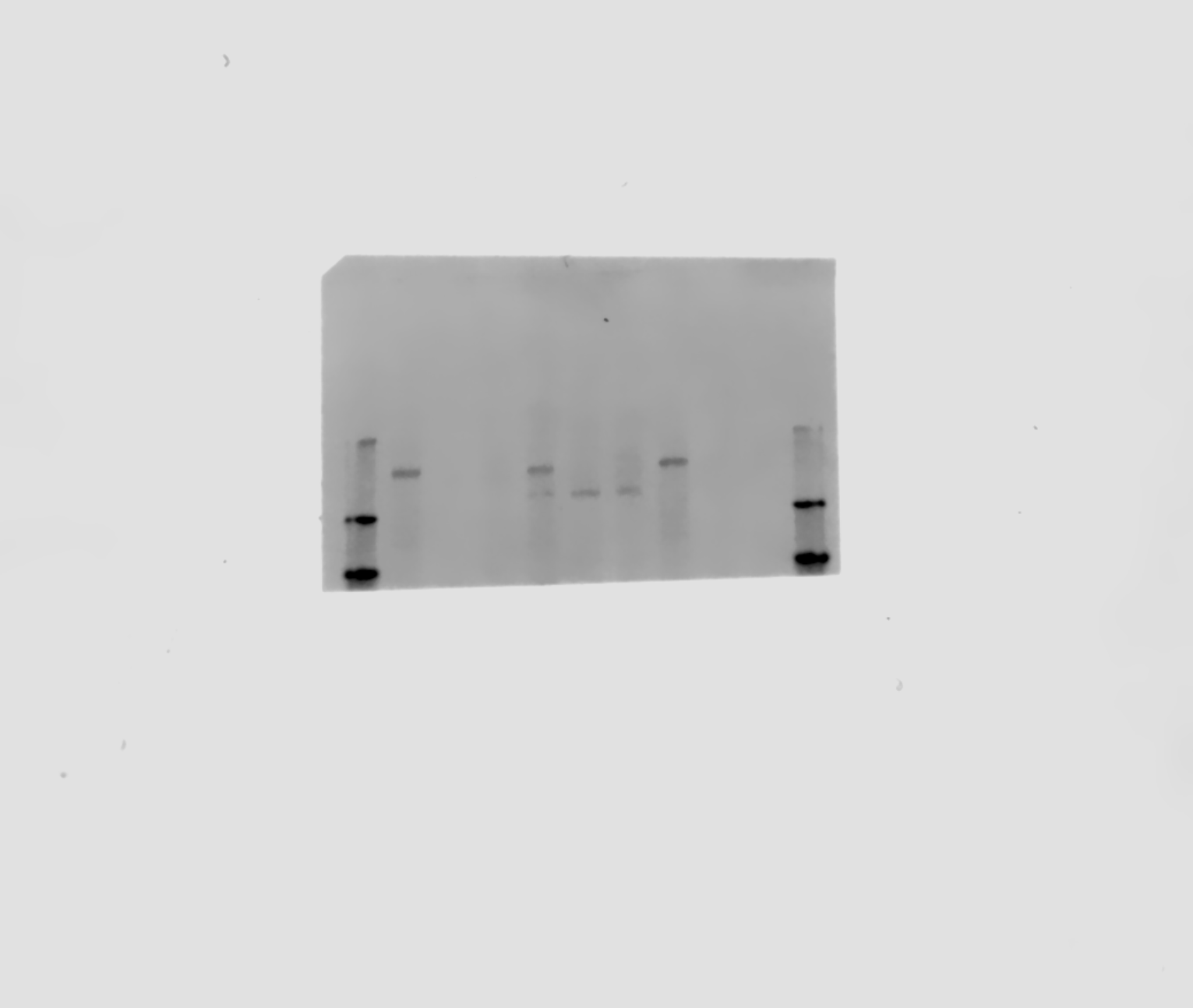

Supplement: Supplementary file 1 [file LSA-2024-03140_SdataF1_F2_F4.4.zip › western blot file-LSA/fig 1B_ flag.tif]

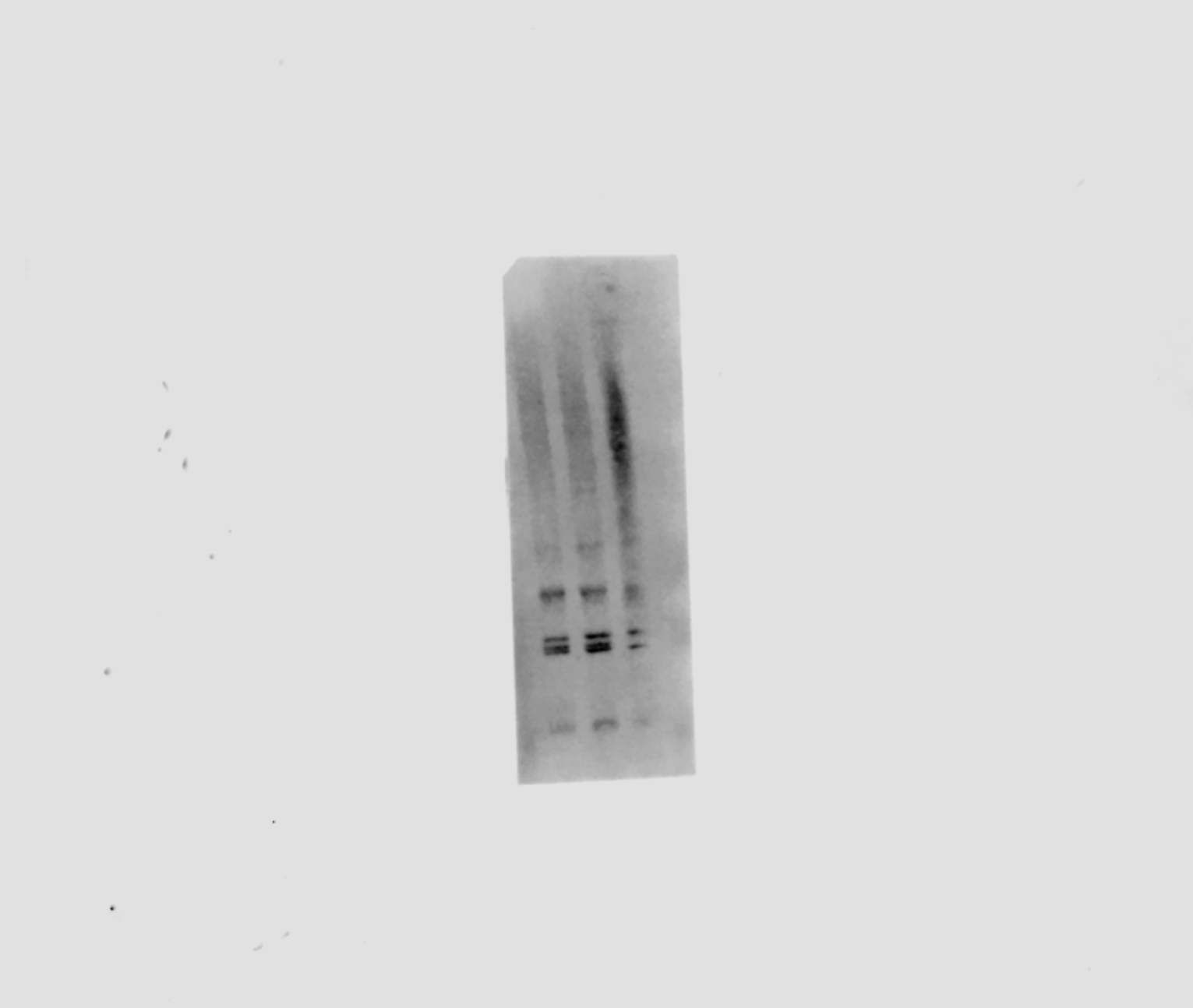

Supplement: Supplementary file 1 [file LSA-2024-03140_SdataF1_F2_F4.4.zip › western blot file-LSA/fig 4 C_his6 .tif]

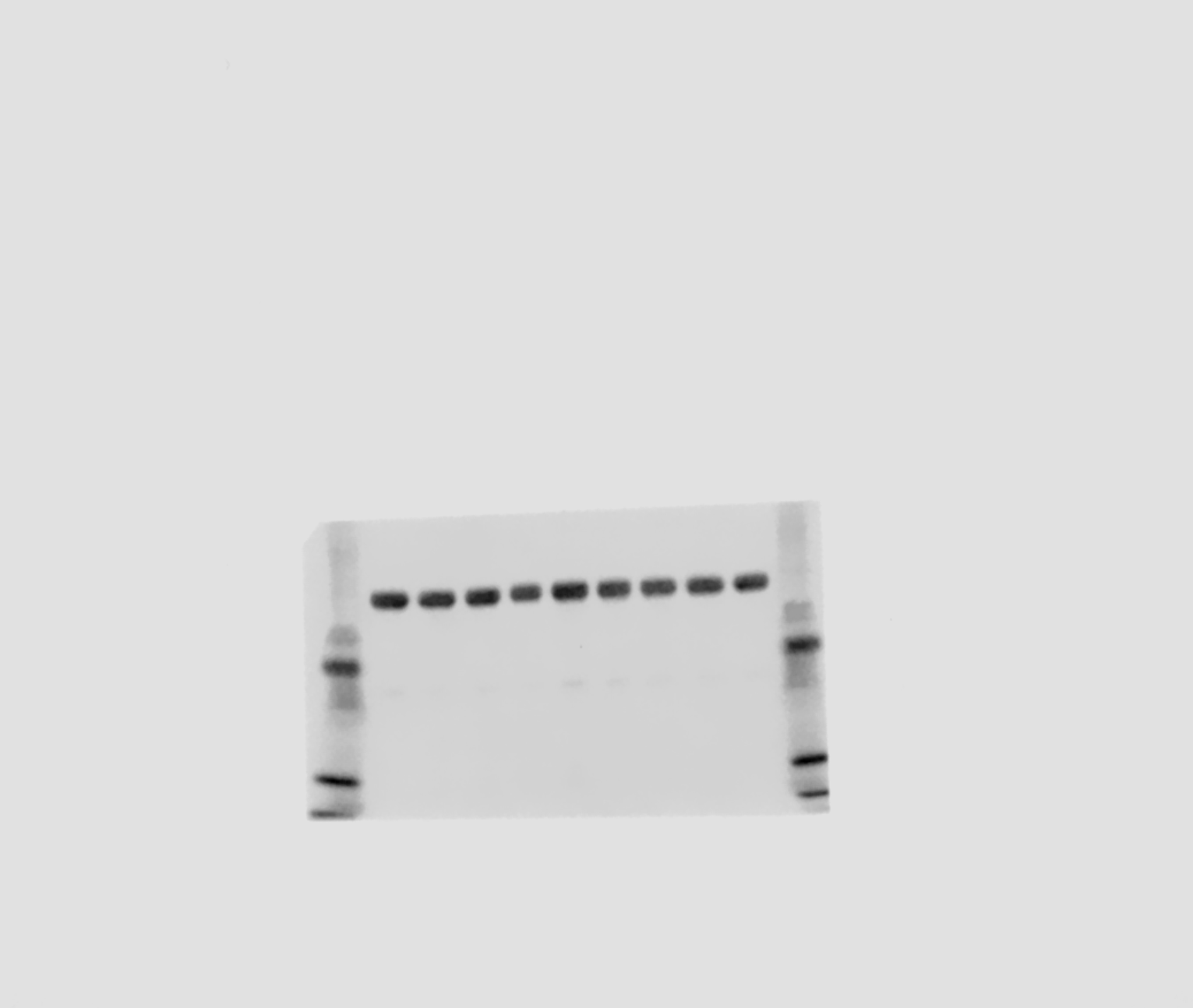

Supplement: Supplementary file 1 [file LSA-2024-03140_SdataF1_F2_F4.4.zip › western blot file-LSA/fig 1B _ tubulin.tif]

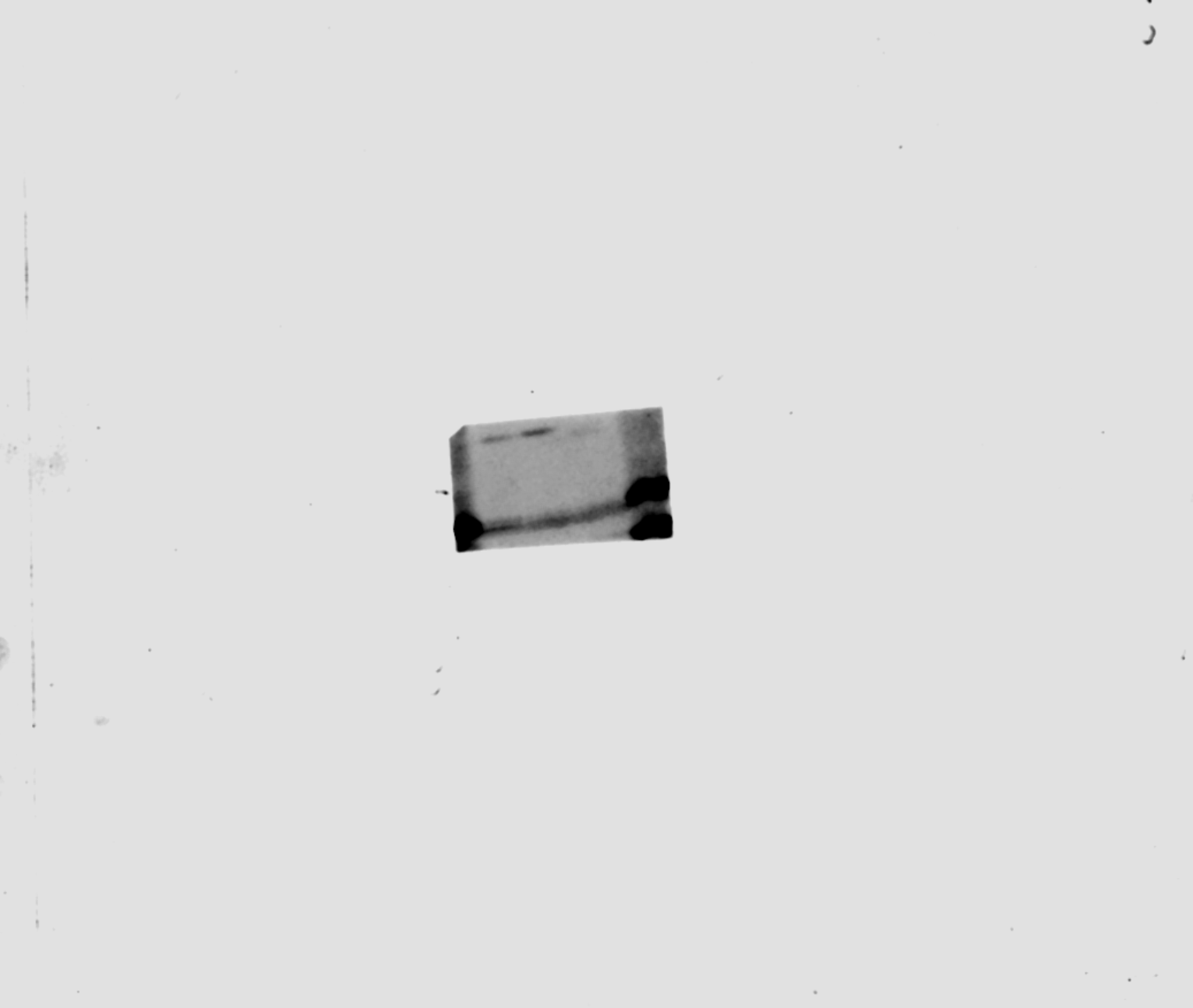

Supplement: Supplementary file 1 [file LSA-2024-03140_SdataF1_F2_F4.4.zip › western blot file-LSA/fig 4C _h2b .tif]

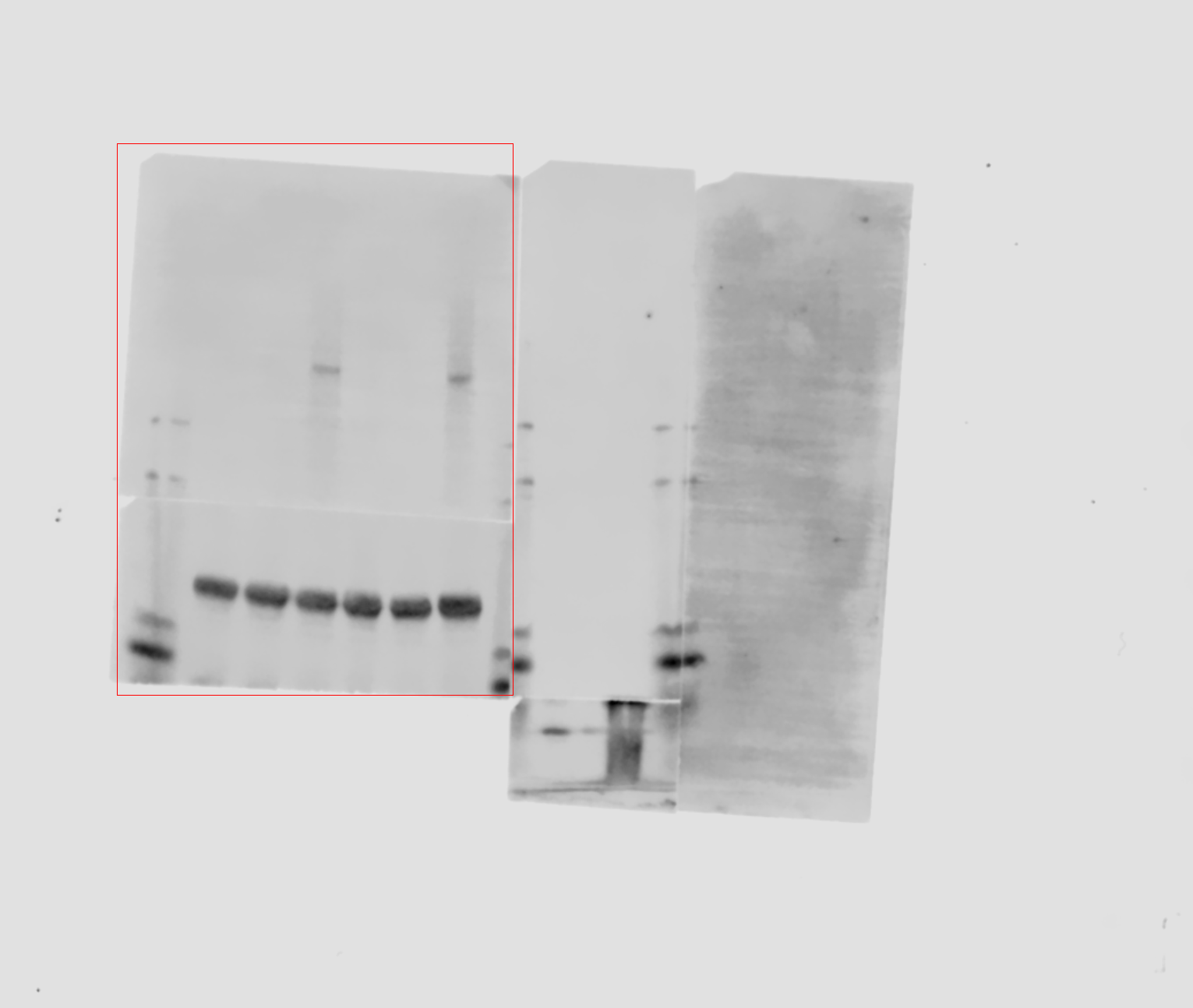

Supplement: Supplementary file 1 [file LSA-2024-03140_SdataF1_F2_F4.4.zip › western blot file-LSA/fig 2A _ mch and tubulin .tif]

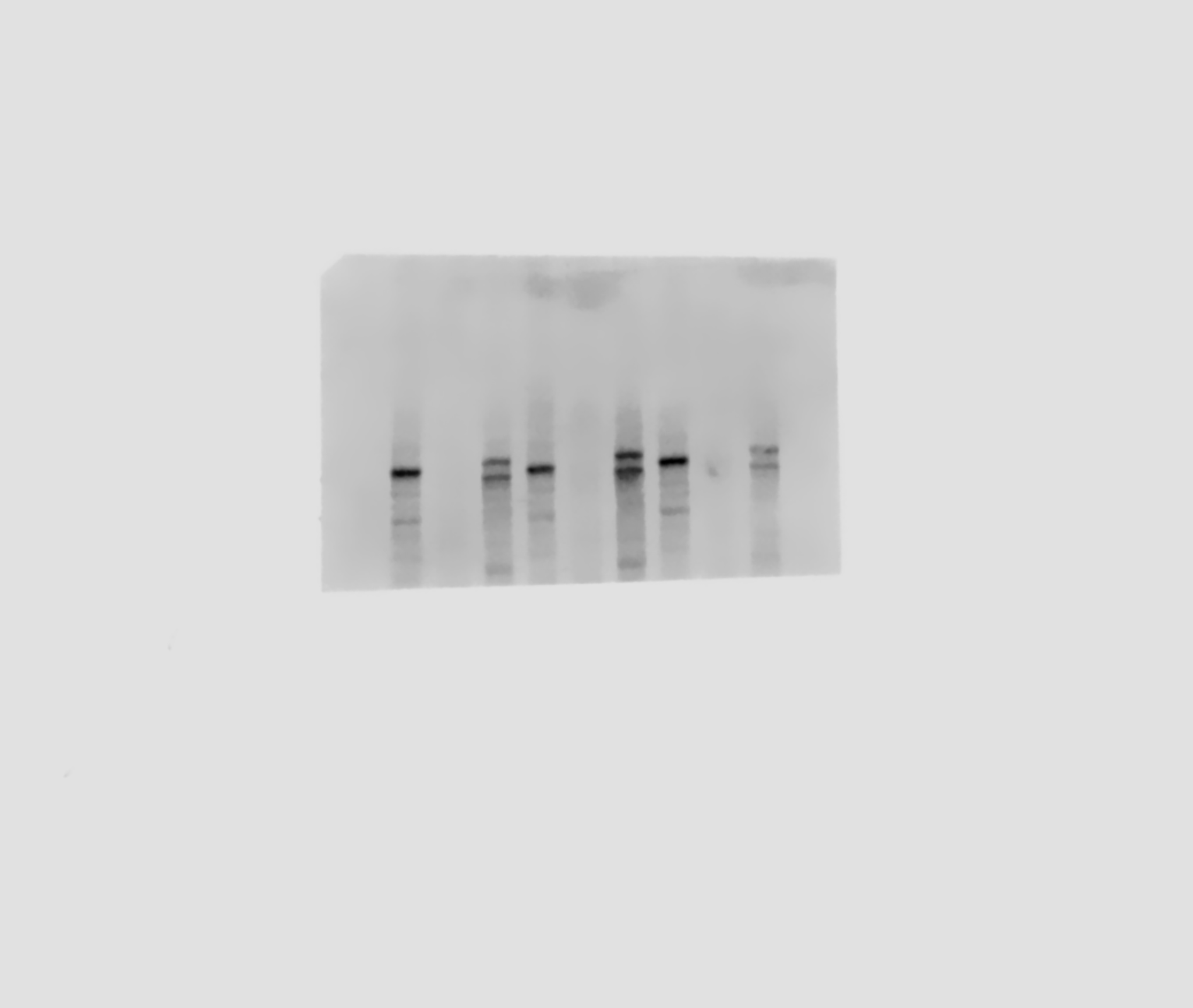

Supplement: Supplementary file 1 [file LSA-2024-03140_SdataF1_F2_F4.4.zip › western blot file-LSA/fig 1B _ pich .tif]
