# Supplementary material for: PICH impacts the spindle assembly checkpoint via its DNA translocase and SUMO-interaction activities
Source: Life Sci Alliance. 2025 Feb 7;8(4):e202403140. doi: 10.26508/lsa.202403140 (PMC11806350; doi:10.26508/lsa.202403140)
Supplement: Supplementary file 5 [file LSA-2024-03140_TableS1.docx]

**Supplementary Table S1**

Sequence of Primers used in this study.

| Primers used for amplification of homology arms | |
| --- | --- |
| H2B Left HA Forward | GTCTTCTTGTTGTCGCGGG |
| H2B Left HA Reverse | CCTTAGCGCTGGTGTAC |
| H2B Right HA Forward | ACAGTGAGTTGGTTGCAAAC |
| H2B Right HA Reverse | GTAGCCGGGCTTGATGG |
| Mad1 Left HA Forward | GATGAGCTTGGGAGTC |
| Mad1 Left HA Reverse | GGCTCCTTTCCTTCCGGGG |
| Mad1 Right HA Forward | ATGGAGGATCTCGGCG |
| Mad1 Right HA Reverse | CTCTTTGGAGCCTGAAG |
| Bub1 Left HA Forward | CCGAGGACGCTGAATAGAAAACTGC |
| Bub1 Left HA Reverse | CAGAGGACGCTGGAAGGCAGCGGC |
| Bub1 Right HA Forward | ATGGACACCCCGGAAAATGTGCTTCAG |
| Bub1 Right HA Reverse | TTGACTGCATTTAACAATCTCGGTCTTC |
| SUMO2 Left HA Forward | TTTAGGCAAGAGTCCGGTGGGAAGC |
| SUMO2 Left HA Reverse | AGTCTCCTCAGCTGCCGCTTCAC |
| SUMO2 Right HA Forward | ATGGCCGACGAAAAGCCC |
| SUMO2 Right HA Reverse | GCGGATTAGGGGTTCGTTGG |
| Haspin Left HA Forward | TCTGGAGCATTTCAGTCTCAAACACCTC |
| Haspin Left HA Reverse | GGACGGCACCTGCCAAGAGGTTC |
| Haspin Right HA Forward | ATGGCAGCTTCTCTCCCAGGACCTGGGAGCCG |
| Haspin Right HA Reverse | GTGTCCTTGGCTCCTCCTGTC |
| gRNA sequences | |
| gRNA H2B | ACTCACTGTTTACTTAGCGC |
| gRNA Mad1 #1 | CAACCATGGAAGACCTGG |
| gRNA Mad1 #2 | ACCATGGTGTTTTCCCCC |
| gRNA Bub1 #1 | GGTTCAGGTTTGGCCGCTGC |
| gRNA Bub1 #2 | ACCAGACGGACACTTACTGA |
| gRNA SUMO2 #1 | GCCCAAGGTGAGCTCGCCGC |
| gRNA SUMO2 #2 | TCGTCGGCCATGGCGAGCGC |
| gRNA Haspin #1 | TTGAACCTCTTGGCGGGTGC |
| gRNA Haspin #2 | GGCCATGGCGGCTTCGCTCC |
| Primers used for confirmation of insertion by genomic PCR | |
| CENP-A F | GAGCCCTCCAAGAGCACCTTG |
| CENP-A R | GCTGTGGTATGGGAGAAAAGGC |
| H2B F | CCCAGCAGCTTGTTGAG |
| H2B R | GCTTATATAGATATACCATAAAGGGAAAATAG |
| Mad1 F | CCATGTTGGCCATTCTGG |
| Mad1 R | CTAATGCGGACAGAACTAC |
| Bub1 F | CTCCACGTCCCCTCTACATGAAGGC |
| Bub1 R | GCACATTAATCTTTAGTAAGTCAAAACAGGACCAGG |
| SUMO2 F | CCGTAGCTAGTCAAAGTCTAGGGTTACCAGC |
| SUMO2 R | CGCGGATTAGGGGTTCGTTGG |
| Haspin F | GGAGTATATCTCCCTCCTGAGGG |
| Haspin R | CCCACCACCAGTTTCCTTTTACAGC |
| Primers used for amplifying DNA fragment | |
| miRFP680 F (with GA linker) | gcaggcgcgggcATGGCGGAAGGCTCC |
| miRFP680 R | CTCTTCCATCACGCCGATC |
| His6-tag/3xFLAG fusion F | CATCATCATTCTTCTGGTgctgactacaaagaccatg |
| His6-tag/3xFLAG fusion R | catggtctttgtagtcagcACCAGAAGAATGATGATG |
| His6-tag 5’ Forward | ATGCACCATCATCATCATCATTC |
| 3xFLAG tag 3’ Reverse | TCCAGCGCCTGCACCAGCCTTGTCATCG |
