# Supplementary material for: PICH impacts the spindle assembly checkpoint via its DNA translocase and SUMO-interaction activities
Source: Life Sci Alliance. 2025 Feb 7;8(4):e202403140. doi: 10.26508/lsa.202403140 (PMC11806350; doi:10.26508/lsa.202403140)
Supplement: Supplementary file 6 [file LSA-2024-03140_TableS2.docx]

**Supplementary table S2**

| REAGENT or RESOURCE | SOURCE | IDENTIFIER |
| --- | --- | --- |
| Antibodies |  |  |
| Mouse anti-FlAG M2 | Millipore/Sigma | Cat# F1804 RRID:AB_262044 |
| Rabbit anti-mCherry | Rockland | Cat# ab167453 RRID:AB_2571870 |
| Rabbit anti-Histone H2A | Cell Signaling Technology | Cat# 12349 **RRID:**AB_2687875 |
| Mouse anti-Histone H2B | Active Motif | Cat# 39210 **RRID:**AB_2793185 |
| Mouse anti alpha tubulin. | Sigma-Aldrich | Cat# T6074, RRID:AB_477582 |
| Mouse anti His 6 | Thermo Fisher Scientific | Cat# MA1-21315, RRID:AB_557403 |
| Mouse anti CENP-A | MBL International | Cat# D115-3, RRID:AB_591074 |
| IRDye^®^ 800CW Goat anti-Rabbit IgG | LI-COR Biosciences | Cat# 926-32211, RRID:AB_621843 |
| IRDye 800CW Goat anti-Mouse IgG | LI-COR Biosciences | Cat# 926-32210, RRID:AB_621842 |
| IRDye 680RD Goat anti-Rabbit IgG | LI-COR Biosciences | Cat# 926-68071, RRID:AB_10956166 |
| IRDye 680RD Goat anti-Mouse IgG | LI-COR Biosciences | Cat# 926-68070, RRID:AB_10956588 |
| Donkey Anti-Rabbit IgG HRP Conjugated | GE healthcare (Cytiva) | Cat# NA934, RRID:AB_772206 |
| Rabbit anti-human PICH | in house | N/A |
| Rabbit anti-SUMO2/3 | in house | N/A |
| Bacterial and Virus Strains |  |  |
| NEB^®^ 5-alpha | New England BioLabs | Cat# C2987 |
| NEB^®^ Turbo | New England BioLabs | Cat# C2984 |
| Chemicals, Peptides, and Recombinant Proteins |  |  |
| Viafect | Promega | Cat# E4981 |
| Doxycycline Hydrochloride | Millipore/Sigma | Cat# D3072 |
| Indole-3-acetic acid (Auxin) | Millipore/Sigma | Cat# I5148 |
| Talon metal affinity resin | Takara | Cat# 635502 |
| mNeonGreen- Trap magnetic agarose beads | Chromotek | Cat# ntma-20, RRID:AB_2827594 |
| Super signal west atto substrate | Thermo Fisher Scientific | Cat# A38554 |
| Critical Commercial Assays |  |  |
| QuikChange II XL Site-Directed Mutagenesis Kit | Agilent | Cat# 200521 |
| Experimental Models: Cell Lines |  |  |
| Human DLD-1 | ATCC | Cat# CCL-221 |
| Oligonucleotides |  |  |
| See table S1 for oligo DNA sequences | IDT | N/A |
| Recombinant DNA |  |  |
| pX330-U6-Chimeric_BB-CBh-hSpCas9 | Addgene | #42230 |
| Software and Algorithms |  |  |
| CRISPR design tools | Zhang laboratory, MIT http://crispr.mit.edu:8079 | N/A |
| CRISPOR | http://crispor.tefor.net | N/A |
| wndchrm | https://github.com/wnd-charm/wnd-charm | N/A |
| Fiji/ImageJ | https://inagej.net/Contributors | N/A |
| Image Studio Version 5.2 | LI-COR | N/A |
| Prism | GraphPad | N/A |
